# A Case of Gallstones Causing Pelvic Pain

**DOI:** 10.1155/2021/5553994

**Published:** 2021-07-19

**Authors:** Aizat Drahman, Kaushi Arulpragasam, Lilach Leibenson, Frank Sardelic

**Affiliations:** ^1^Department of Surgery, Royal Prince Alfred Hospital, 2050 Sydney, New South Wales, Australia; ^2^Department of Obstetrics and Gynaecology, King Edward Memorial Hospital, 6008 Subiaco, Western Australia, Australia; ^3^Department of Surgery, Tamworth Base Hospital, 2340 Tamworth, New South Wales, Australia

## Abstract

**Introduction:**

Assessing abdominal pain, particularly in women of reproductive age, requires thorough history taking, clinical examination, and investigations to obtain an accurate diagnosis. Both surgical and gynecological causes need to be considered, particularly previous relevant surgical history. *Presentation of case.* We report a case of pelvic pain secondary to multiple gallstones found within the pelvic cavity postlaparoscopic cholecystectomy. Thorough investigations have been conducted without any obvious cause found. The pain was debilitating and largely affecting the patient's quality of life. Therefore, decision to perform diagnostic laparoscopy and gallstones was found all over pelvic cavity and retrieved. Her pain resolved post operatively.

**Conclusions:**

Gallbladder perforation and stone spillage are the most common complications of laparoscopic cholecystectomy that arise during the removal and dissection of gallbladder and can cause significant morbidity if not managed early, especially retrieval of the stones intraoperatively. Therefore, patient with history of previous cholecystectomy with stone spillage presenting with undifferentiated abdominal pain and early diagnostic laparoscopy should be considered.

## 1. Introduction

The decision to perform early diagnostic laparoscopy on a patient presenting with undifferentiated abdominal pain must be considered early, excluding life-threatening differentials and weighing overall risks and benefits. Gallbladder perforation and spillage of stones in the gallbladder are most common complications of laparoscopic cholecystectomy. Most reported cases of stones spillage were in patients with challenging biliary anatomy and morbidly obese patients [1]. Most importantly, decision to remove the spillage of gallstones intraoperatively will prevent complications in the future.

## 2. Case Presentation

**Figure 1:**
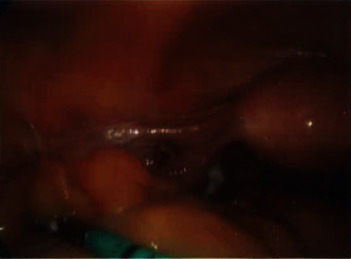
Stone-like material deeply embedded in broad ligament.

**Figure 2:**
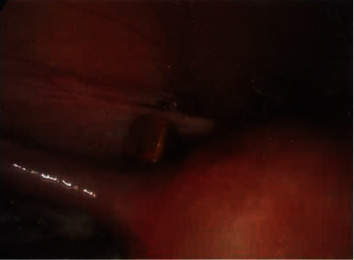
Stone-like material deeply embedded in peritoneum.

**Figure 3:**
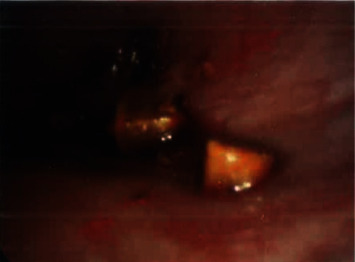
Deeply embedded multiple fragments of stone-like material in peritoneal cavity.

A 38-year-old nonpregnant obese female presented to the Emergency Department after being referred by her general practitioner, with a 3-day history of severe sharp, cyclical pelvic pain which lasted seven days with no exacerbating or relieving factors. She had normal menstrual flow and denied any history of dyspareunia, abnormal vaginal discharge, nausea, vomiting, altered bowel habit, or urinary symptoms. Her obstetric history included three vaginal deliveries, and she was up to date with her pap smears. Her past surgical history included a laparoscopic appendicectomy and cholecystectomy performed 2 months ago overseas in Thailand due to concurrent appendicitis and cholecystitis. She did not have any other significant medical history. She smoked 10 cigarettes a day and was in a stable relationship with her long-term partner. On examination, she appeared generally well and has a body mass index of 36. Abdominal and vaginal examination (done by gynaecology team) demonstrated generalized lower abdominal tenderness with no peritonism and palpable masses. CT abdomen/pelvis and ultrasound of the pelvis were essentially normal with no identifiable cause for her pain. As the pain was debilitating and largely affecting her quality of life, a decision was taken to carry out a diagnostic laparoscopy. Intraoperatively, the uterus, fallopian tubes, and ovaries appeared grossly normal with no evidence of endometriosis. Multiple “stone-like” deposits were found deeply embedded over the uterovesical pouch and left broad ligament (Figures 1–3). The “stone-likë” deposits were meticulously retrieved using fine nontoothed graspers. The deposits disintegrated easily with minimal force. We concluded the laparoscopy by doing pelvic peritoneal washout. The operation was uncomplicated. The following day, the patient was discharged home. She was followed up in clinic 3 weeks later reporting no further episodes of pain. Histopathology confirmed the “stone-like” deposits to be gallstones made of cholesterol. A retrospective review of her operation report from Thailand (which was obtained 1 week after her diagnostic laparoscopy) was highlighted during removal of gallbladder, and it impacted into the abdominal wound. An attempt to decompress the gallbladder with an endoscopic suction caused the gallbladder wall to perforate, resulting in accidental spillage of gallstones into the peritoneal cavity. She was informed by the surgeon of the complications postoperatively but upon further questioning, she was not aware of the severity of the complications.

## 3. Discussion

A literature review demonstrated several cases of gallstone-related pelvic pain post gallbladder perforation during cholecystectomy. Symptoms include severe abdominal pain, fever, and anorexia [1]. Gallbladder perforation and stone spillage are the most common complications of laparoscopic cholecystectomy that arise during the removal and dissection of gallbladder [1–3]. In fact, the percentage recorded was 10-40% for gallbladder perforation and 6-30% for stone spillage [4, 5]. The mean time of patients having laparoscopic cholecystectomy and reported complications mainly abdominal pain caused by stone spillage is reported to be 10.4 months, with a range of 10 days to 20 years [6–8]. It is recommended that gallbladder wall perforation is best dealt immediately during surgery, by extracting as much visible stones as possible and with copious irrigation with normal saline of the peritoneal cavity, which shown to reduce bacterial contamination as well as providing a fluid-gas interface to allow gallstones to float, hence facilitate stone retrieval [2, 7, 9, 10]. 20% of dropped gallstones are not removed because of their location, number, and fragmentation. Therefore, most surgeons believe that free intraperitoneal stones are not a justification for a laparotomy [2, 3]. Another complicating factor is most bile stones that are radiolucent on CT abdomen and pelvis (only 5% visible as compared to 98% on ultrasound), making diagnosis more challenging in patients with undifferentiated abdominal pain [3]. However, 0.08-7% of patients can develop major complications of unretrieved peritoneal gallstones [4, 5]. Complications include infection or abscess, adhesions, fibrosis, cutaneous sinuses, inflammation, small bowel obstruction, or generalized septicemia [3, 11–15]. In this case, the presenting problem was mainly debilitating pain without any evidence of other major complications. In retrospect, the best practice was to chase her operation report of her previous procedures, but this will delay her treatment.

## 4. Conclusions

Diagnostic laparoscopy in patients presenting with undifferentiated abdominal pain should be considered early. In addition, another consideration for early diagnostic laparoscopy is when there is history of previous surgical complications, in this case, gallbladder perforation and spillage of stones. Therefore, gallstone's spillage intraoperatively, if not retrieved, can cause significant morbidity to the patient in the future and should be advised of the potential complications immediately postoperatively.

## Data Availability

No data were used to support this study.
